# Residual Utilization of Crab Solid Parts for Powder Production and Application as a Structural Component in the Polymeric Matrix of Biodegradable Films

**DOI:** 10.3390/polym17243334

**Published:** 2025-12-17

**Authors:** Fábio G. Teles, Railene H. C. R. Araújo, Aline D. B. Arriel, Valdilene M. C. Soares, Adriano S. Silva, Kalinny A. Alves, Maria A. S. Morais, Patrícia L. D. Morais, Nayara S. Rocha, Antonio G. B. Lima, João M. P. Q. Delgado

**Affiliations:** 1 Department of Food Engineering, Federal University of Campina Grande, Campina Grande 58429-900, Paraíba, Brazil; ftpesca2019@gmail.com (F.G.T.); aline.barbosa@estudante.ufcg.edu.br (A.D.B.A.); valdilene.maria@estudante.ufcg.edu.br (V.M.C.S.); adriano.sant@professor.ufcg.edu.br (A.S.S.); nayara.santos@estudante.ufcg.edu.br (N.S.R.); 2 Department of Process Engineering, Federal University of Campina Grande, Campina Grande 58429-900, Paraíba, Brazil; kalialves1607@gmail.com; 3 Department of Agronomic and Forest Sciences, Federal Rural University of the Semi-Arid Region, Mossoró 59625-900, Rio Grande do Norte, Brazil; aparecida8sm@gmail.com (M.A.S.M.); patricia.morais@ufersa.edu.br (P.L.D.M.); 4 Department of Mechanical Engineering, Federal University of Campina Grande, Campina Grande 58429-900, Paraíba, Brazil; antonio.gilson@ufcg.edu.br; 5 CONSTRUCT-BPG, Department of Civil and Georesources Engineering, Faculty of Engineering, University of Porto, 4200-465 Porto, Portugal

**Keywords:** packaging, mechanical properties, marine residues, natural polymers, *Ucides cordatus*

## Abstract

Natural fillers have been widely explored to enhance the mechanical and barrier properties of biodegradable films. In this study, a mineral-rich powder obtained from the solid components of *Ucides cordatus* crab shells was processed (washing, drying, milling, and sieving at 75 µm) and extensively characterized using SEM, FTIR XRD, EDX, mineral analysis, hygroscopicity, density, and particle size distribution. The powder exhibited heterogeneous morphology and contained 22.52 g·kg^−1^ of calcium carbonate, along with other trace minerals; its crystalline profile indicated the presence of both calcite and aragonite. Low hygroscopicity (1.76%) and a true density of 2.11 g/cm^3^ were also observed. When incorporated into pectin-based films at 1–5%, the filler promoted a reduction in film thickness, indicating enhanced structural compaction. Solubility increased linearly with filler content, whereas water vapor permeability (WVP) decreased at 1% and 2% but rose again at 4% and 5%, correlating positively with solubility (r = 0.895). Films containing 4% and 5% exhibited higher tensile strength and elastic modulus, confirming increased rigidity. At elevated concentrations, the films also became less luminous and more chromatic. Overall, the findings demonstrate that crab-shell mineral powder is a viable and sustainable reinforcement capable of tailoring the structural, mechanical, and barrier performance of biodegradable films.

## 1. Introduction

The rapid expansion of the aquatic organism processing industry has led, in recent years, to the generation of substantial volumes of solid waste, particularly those originating from crustacean processing, such as crab residues. Globally, the annual production of this waste is estimated at approximately 6 to 8 million tons [1], representing not only an environmental liability but also a byproduct rich in industrially relevant biomolecules, including proteins [2], chitin [3], minerals [4] and pigments [5]. This biochemical composition confers high valorization potential to the residue, fostering the development of new products and contributing to the overall sustainability of the sector by mitigating the environmental impacts associated with improper disposal [5].

In this context, there is growing interest in the development of biodegradable films derived from renewable resources and food industry byproducts as more sustainable alternatives to conventional packaging materials [6]. Concurrently, recent advances have demonstrated that the performance of such films can be enhanced through the incorporation of natural fillers capable of inducing structural modifications, thereby increasing mechanical strength and modulating diffusion processes [7]. Accordingly, the powder obtained from crab-processing residues emerges as a promising candidate for application in biodegradable film formulations.

Its mineral-rich composition, especially the high calcium carbonate (CaCO_3_) content, can modify the structure of the composite by enhancing the mechanical properties of the films [4], increasing stiffness, elastic modulus, opacity and, in some cases, barrier performance, provided that adequate dispersion and compatibility with the polymer matrix are achieved [8]. Moreover, morphological and textural characteristics may support interlocking with the polymer matrix, reinforcing the structural integrity of the film [9]. Likewise, the presence of functional groups associated with chitin and proteins can enhance interfacial adhesion between the filler and the matrix [2,3], while the predominance of an organized crystalline phase provides the material with density and structural arrangement that directly influence the physicomechanical properties of the films [4,9].

Among the most promising alternatives for the production of biodegradable films is the combination of polysaccharides and essential oils, which enables the creation of materials with complementary structural and functional properties. In this regard, the matrix selected for production of the films proposed in this study is composed of pectin and pomegranate seed essential oil. Pectin is a natural, biodegradable, non-toxic, and edible biopolymer that is highly versatile for film formation and exhibits biocompatibility with additives or other polymers [10]. The essential oil derived from pomegranate seeds may function as a bioactive compound, conferring potential antioxidant and antibacterial attributes to the formulation [11,12].

Nonetheless, taking into account the particle-size distribution of the powder, its mineral-organic composition, stiffness and density, increased filler concentrations may promote the development of aggregates that could undermine the physical and mechanical properties of the films [13]. Thus, it is necessary to establish a balance between the structural integrity provided by the filler and the preservation of the film’s functionality.

Therefore, conducting tests with different filler levels is essential for evaluating the effect of each concentration on the structural and functional properties of the films. A reduction in porosity and molecular mobility typically results from a uniform distribution of powder particles within the matrix, which can positively affect physical and mechanical performance [14,15].

Accordingly, the objective of this study was to employ whole crab shell powder as a structural component in a pectin/essential oil matrix, evaluating different filler concentrations (0%, 1%, 2%, 3%, 4%, and 5%) and their effects on the physical and mechanical properties of the films. To achieve this, the powder was characterized in terms of its morphology, chemical composition and structural features.

## 2. Materials and Methods

### 2.1. Materials Acquisition

For the composition of the polymeric matrix, pomegranate seed oil, citric pectin, glycerol and Polysorbate Tween 80 were used, with or without the addition of powder extracted from the solid parts of the crab. The ‘Mollar’ pomegranate seed oil was extracted from fully ripe fruits harvested from a commercial orchard, using anhydrous and hydrated ethanol (90 °GL) in a Soxhlet-type extractor, according to the methodology described by Oliveira et al. [16]. High-methoxyl citrus pectin ATM (Adicel, Belo Horizonte, Brazil) was commercially purchased, as were the glycerol (Uniphar, Anápolis, Brazil) and Polysorbate Tween 80 (Exodo Científica, Sumaré, Brazil).

### 2.2. Process for Obtaining the Solid Parts of the Crab

Forty crabs (*Ucides cordatus*) were collected from a mangrove area located on the coast of Joao Pessoa, PB-Brazil and transported to the laboratory of the Federal University of Campina Grande, PB, situated 128.6 km from the collection site.

**Figure 1:**
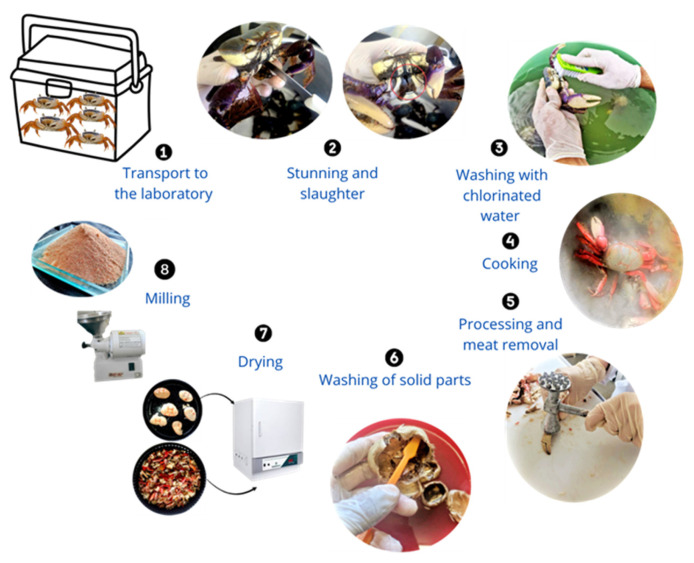
Illustrated steps of the processing of *Ucides cordatus* crabs for obtaining powder from the solid parts.

In the laboratory (see Figure 1), the animals were anesthetized in chlorinated water at 5 ppm with the addition of ice, maintaining a temperature of 4 °C for 8 min to reduce motor activity prior to slaughter. After euthanasia, the crabs were rinsed with chlorinated water to remove all residues (Step 3). Subsequently, the specimens were boiled at 100 °C for 15 min and then subjected to a thermal shock by immersion in water at 2 °C for 10 min (with ice) to facilitate the separation of the meat from solid fractions (Step 4). The solid fractions, carapace and legs, were removed and collected (Step 5), then submitted to a second wash in cold chlorinated water (Step 6). The washed solids were dried at 100 °C for 24 h in a recirculating-air oven (Quimis Q314M242, São Paulo, Brazil), following the protocol described by Kaewprachu & Jaisan [17] (Step 7). Finally, the dried material was milled using a hammer mill (Botini B5509, Botini, Paraná, Brazil) to convert the solid residues into powder (Step 8).

### 2.3. Powder Characterization

The powder used in the film formulation was sieved through a 200-mesh screen (75 µm) according to the methodology described by Alípio et al. [18], and subsequently characterized by scanning electron microscopy (SEM), Fourier transform infrared spectroscopy (FTIR), hygroscopicity, X-ray diffraction (XRD), energy-dispersive X-ray spectroscopy (EDX), true density determination, quantitative analysis of absolute mineral content and particle size. All analyses were performed using a powder sample previously sieved through the 75 µm mesh, which was the same one used in the film fabrication.

#### 2.3.1. Scanning Electron Microscopy (SEM)

A Tescan VEGA 3 LMU microscope (Tescan, Brno-Kohoutovice, Czech Republic) to look at the crab powder’s surface morphology with scanning electron microscopy (SEM). To make sure they stuck properly, the samples were attached to metal stubs with conductive carbon tape. Micrographs were captured at magnifications between 35× and 1600×.

#### 2.3.2. Fourier Transform Infrared Spectroscopy (FTIR)

A spectrometer (Vertex 70, Bruker, Karlsruhe, Germany) was used, operating in absorbance mode with a spectral resolution of 4 cm^−1^, 64 scan accumulations and a wavelength range from 4000 to 500 cm^−1^.

#### 2.3.3. Hygroscopicity

The methodology followed the protocol described by Huerta-Vera et al. [19], with adaptations. One gram of the sample was weighed and transferred to Petri dishes, which were then placed in a hermetically sealed container containing a saturated sodium chloride (NaCl) solution. The saturated NaCl solution created a controlled relative humidity environment of approximately 75% at 25 °C. Hygroscopicity was determined using Equation (1). The samples were removed from the container and weighed.


(1) $$Hygroscopicity\;(\%)=\frac{Water\;absorbed\;(g)}{Sample\;weight\;(g)}\;\times \;100$$

#### 2.3.4. X-Ray Diffraction (XRD)

The samples were characterized by X-ray diffraction using an X-ray diffractometer (D2 Phaser, Bruker, Karlsruhe, Germany) with Cu Kα radiation, operating at 40 kV and 30 mA. Measurements were performed in continuous scan mode over a 2θ range from 5° to 50°, with a step size of 0.02° and a predefined counting time of 1.20 s per step.

#### 2.3.5. Energy-Dispersive X-Ray Spectroscopy (EDX)

The semiquantitative analysis of the elements present in the samples was performed by energy-dispersive X-ray spectroscopy (EDX) using a spectrometer (EDX-720, Shimadzu, Kyoto, Japan). The sample was pressed at 10 tons for 2 min to form a pallet with a diameter of 2 mm.

#### 2.3.6. True Density

The true density was determined by helium gas pycnometry using a pycnometer (Upyc 1200e v5.04 Pycnometer, Quantachrome Corporation, Boynton Beach, FL, USA) operating with helium gas (He) at 1.4 bar. A 1.25 g sample was weighed and analyzed through ten automatic runs. The density values were expressed in g·cm^−3^ after stabilization of the readings and according to the instrument’s repeatability criteria. The results were reported as mean ± standard deviation.

#### 2.3.7. Quantitative Analysis of Absolute Mineral Content

The determination of phosphorus, potassium, calcium, magnesium, copper, iron, manganese, zinc and sodium contents in the crab powder was performed according to an adapted protocol from the Manual of Chemical Analysis of Plant Tissue [20]. The process involved wet digestion (nitric-perchloric) of powder samples (0.5 g). 4.0 mL of pure HNO_3_ were added, and the mixture was left to stand for approximately 12 h (pre-digestion). The mixture was then slowly heated to 120 °C until NO_2_ emission ceased and the acid volume was reduced by half. Next, 2.0 mL of HClO_4_ were added, and the mixture was heated to approximately 180 °C. After cooling, the solution was transferred to a 25 mL volumetric flask, the volume was adjusted with ultrapure water, and filtration was performed when necessary to remove insoluble particles. The obtained extracts were used to quantify the elements according to the official methods of embrapa: phosphorus was measured by colorimetry (formation of the phospho-molybdate/vanadate complex); potassium and sodium were determined by flame emission photometry; and calcium, magnesium, copper, iron, manganese and zinc were quantified by atomic absorption spectrometry (AAS), using certified metal standards and lanthanum as corrective agent in cases of interference.

#### 2.3.8. Particle Size

The particle size distribution and mean particle diameter were determined from images obtained by scanning electron microscopy (SEM) at a magnification of 35×. For this purpose, the ImageJ v. 1.54k software was used to process the micrographs, enabling the measurement of particle size. Subsequently, the data were analyzed with the Origin software to generate the particle size distribution plot. A total of 150 measurements were performed according to the protocol described by Regadas et al. [21].

### 2.4. Film Characterization

#### 2.4.1. Preparation of Film-Forming Solutions

The film-forming solutions were prepared according to the methodology described by Alves et al. [22], with minor modifications. At this stage, an experiment was conducted under a completely randomized design, with four replicates, to evaluate the effect of adding different concentrations of crab powder, as well as its function as a structuring agent in film formation. Six distinct films were developed during this process, each incorporating different powder concentrations into the polymeric matrix, specifically 0, 1, 2, 3, 4, and 5% (*w*/*w*).

**Figure 2:**
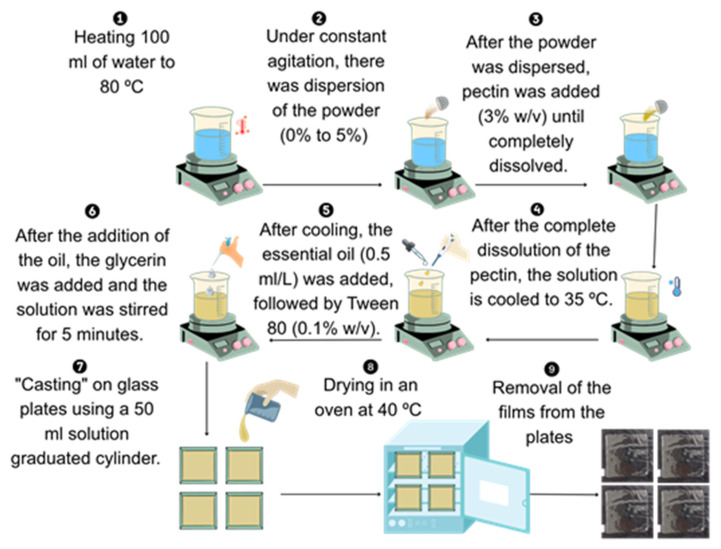
Illustrated flowchart of the film fabrication process.

The film-forming process began by heating 200 mL of distilled water to 80 °C under constant stirring. The crab-shell powder was then gradually added, followed by the addition of citrus pectin at 6.0% (*w*/*v*). Once the pectin was fully dissolved and under constant agitation, the temperature of the mixture was reduced to 35 °C. Then, 0.05 mL·L^−1^ of pomegranate seed oil and 0.1% (*w*/*v*) Tween 80 were added. Glycerol was subsequently incorporated at 40% (*w*/*w*) relative to the mass of pectin and mixture was stirred for an additional 5 min to ensure proper homogenization of all components. The final solution was then poured onto glass plates (14 × 14 cm), dispensing 50 mL per plate using a graduated cylinder, in quadruplicate. The films were dried in a forced-air oven (Marconi, MA 035/3IN250, São Paulo, Brazil) at 40 °C for 24 h (Figure 2).

#### 2.4.2. Scanning Electron Microscopy (SEM)

The morphological analysis of the films was performed by SEM using a TESCAN VEGA S514 microscope (Brno-Kohoutovice, Czech Republic). For surface analysis, the samples were cut into circular pieces with a diameter of 15 mm, and for cross-sectional analysis, they were cut into rectangular pieces measuring 10 × 15 mm. Samples were taken from the central region of the films in triplicate, excluding the edges. To improve the electrical conductivity of the samples prior to imaging, they were sputter-coated with a thin layer of gold. Surface images were obtained at a magnification of 2000×, and detailed cross-sectional images were obtained at 5000× magnification. The samples were mounted on stubs using conductive carbon tape and examined under high vacuum to assess particle dispersion and the presence of structural defects.

#### 2.4.3. Thickness

The thickness of the films was determined using a manual external micrometer (110.284-NEW, Digimess, São Paulo, Brazil) with a precision of 0.01 mm. Measurements were taken at four locations: one at the center of the film and three equidistant points near the edges. All measurements were performed in quadruplicate using film samples with dimensions of 6.5 × 3 cm.

#### 2.4.4. Solubility

The solubility of the films was determined following a methodology adapted from Alves et al. [22]. For this purpose, samples were prepared in quadruplicate, cut into dimensions of 2 × 2 cm, and subsequently weighed. First, the films were dried in a forced-air oven at 105 °C for 24 h to obtain the initial dry mass. The dried samples were then immersed in 30 mL of distilled water at 25 °C, where they remained for 24 h. After immersion, the films were dried again under the same conditions to determine the final mass. Solubility was expressed as the percentage of mass loss relative to the original dry mass.

#### 2.4.5. Color Attributes

The color characterization of the films was performed using a colorimeter (Chroma Meter CR-400, Konica Minolta, Ramsey, NJ, USA), operating within the CIE L*, a* and b* coordinate system. Measurements were taken over a white paper background to ensure uniform reflectance. Based on the obtained L*, a* and b* values, the hue angle (°h) and chroma saturations (C*) were calculated according to Equations (2) and (3), respectively.


(2) $${}^{\circ}h=\mathrm{arc}\tan\left(\frac{b*}{a*}\right)$$

(3) $$C*\;=\;\sqrt{a^{2}+b^{2}}$$

#### 2.4.6. Water Vapor Permeability

Water Vapor Permeability (WVP) was determined using the gravimetric method in accordance with ASTM E96/E96M-16 [23] and following the methodology described by Alves et al. [22]. The films were the placed onto circular glass capsules with a diameter of 4 cm, which contained dried silica gel inside, representing 0% relative humidity. The capsules were subsequently placed in desiccators at 25 °C containing a saturated NaCl solution, resulting in a relative humidity of approximately 75% around the capsules. The samples were weighed every 24 h for up to seven days, or until a 4% mass gain of the silica was observed. Analyses were conducted in quadruplicate. Additionally, a control specimen (without film), containing only silica, was exclusively attributable to vapor permeation through the films. WVP was calculated according to Equation (4):

(4) $$WVP=\frac{AW\;\times \;FT}{S\;\times \;\Delta P\;\times \;RHD}$$

where AW is the weight loss of the film per hour (g·h^−1^); FT is the film thickness (mm); S is the exposed film area (m^2^); ΔP is the tabulated water vapor pressure (kPa) at a 25 °C; and RHD is the relative humidity difference (initial relative humidity minus the average relative humidity over the seven-day measurement period).

#### 2.4.7. Tensile Strength

The tensile strength of the films was evaluated in eight replicates using a TA.XT. plus texture analyzer (Stable Micro Systems Ltd., Vienna Court, UK). According to ASTM D-882-12 [24], the samples were cut into strips measuring 6.5 cm in length and 3 cm in width. The system was calibrated using the Texture Expert software, with a return distance set to 20 mm. The tests were conducted at a steady tensile rate of 0.21 mm, utilizing a load cell rated for 500 N. From the gathered data, the mechanical properties were analyzed, such as tensile strength, elongation, toughness, and elastic modulus.

#### 2.4.8. Photographic Images of the Films

The photographic images of the films were captured using a Nikon COOlPIX P530 camera Nikon Corporation, Manaus, Brazil). The photographs were taken from film samples measuring 6.5 cm × 8 cm.

### 2.5. Statistical Analysis

For the powder’s physical properties and the relationship between water vapor permeability (WVP) and solubility, Pearson correlation analyses were performed using R software, version 4.5.1. Correlation coefficients ® and significance levels (p) were calculated for each pair of variables under investigation. Film characterization data were subjected to analysis of variance (ANOVA) and when statistically significant differences were observed, Tukey’s honestly significant difference (HSD) test (*p* ≤ 0.05) was applied for multiple mean comparisons. In addition to ANOVA, regression analyses were conducted to assess the influence of powder loading on the observed film responses. Both first-order (linear) and second-order (quadratic) regressions models were fitted for each measured property, with the significance of the regression coefficients (β_1_ e β_2_) and the coefficient as determination (R^2^) serving as criteria for selecting the most suitable model. All statistical analyses, including ANOVA, regression and Tukey’s HSD test, were performed using SISVAR version 5.6 [25].

## 3. Results and Discussion

### 3.1. Powder Characterization Results

#### 3.1.1. Scanning Electron Microscopy (SEM)

**Figure 3:**
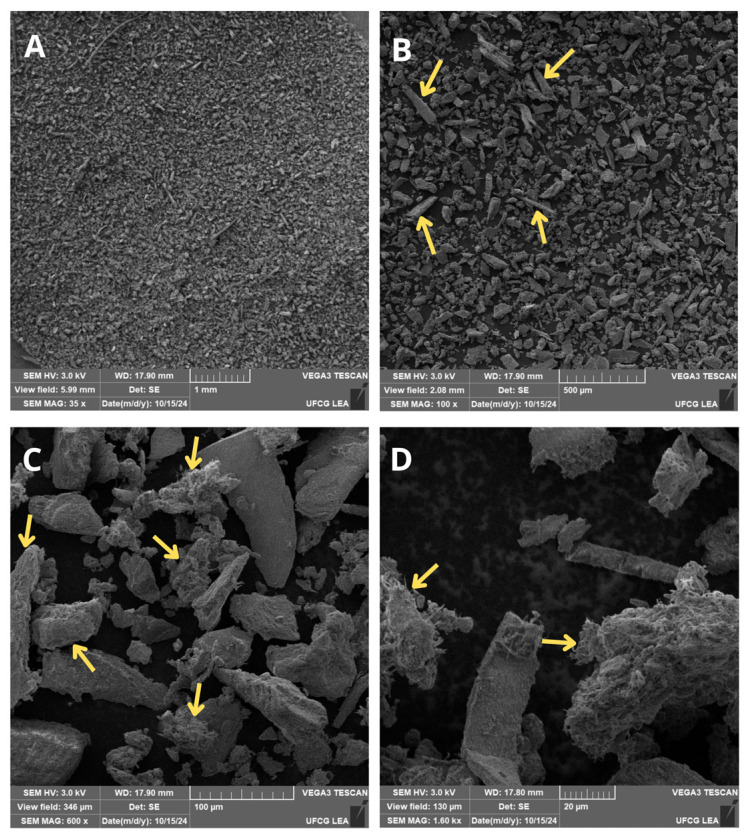
SEM micrographs of the surface of the crab shell powder particles at different magnifications: (**A**) 35×, (**B**) 100×, (**C**) 600×, and (**D**) 1600×.

It can be observed from the SEM micrographs that the powder exhibits a heterogeneous morphology, characterized by particles of varying sizes and irregular shapes (Figure 3A). At this magnification, some particles display relatively smoot surfaces, whereas others present more pronounced angular fragmentation. At higher magnifications, it is possible to observe crystalline particles with smooth, nodular-like surfaces, as well as elongated structures resembling rod-shaped elements, in addition to smaller particles with non-spherical morphologies (Figure 3B). The micrographs also show rough and filamentous textures (Figure 3C,D), which means that the material is structurally complex. Nwaeju et al. [9] also reported similar results; they identified morphological heterogeneity in materials derived from the shell crab species. These features described in the SEM analysis are, in fact, characteristic of chitinous materials, whether originating from crustaceans [26] or insects [27].

These characteristics may be fundamental for interfacial adhesion with polymeric matrices, as they can influence the contact area and promote physical anchoring between the particles and polymer matrix [28]. This kind of behavior makes it easier for stress to move from one part of a composite to another, which is shown by the composites’ mechanical performance, especially their ability to resist breaking [29]. Moreover, these properties are directly related to both particle size and filler loading percentage employed [30]. The rough and filamentous reported by Zhang et al. [31], rougher surfaces reduce the energy barriers governing interactions with liquid phases, thereby affecting water absorption.

#### 3.1.2. Fourier Transform Infrared Spectroscopy (FT-IR)

**Figure 4:**
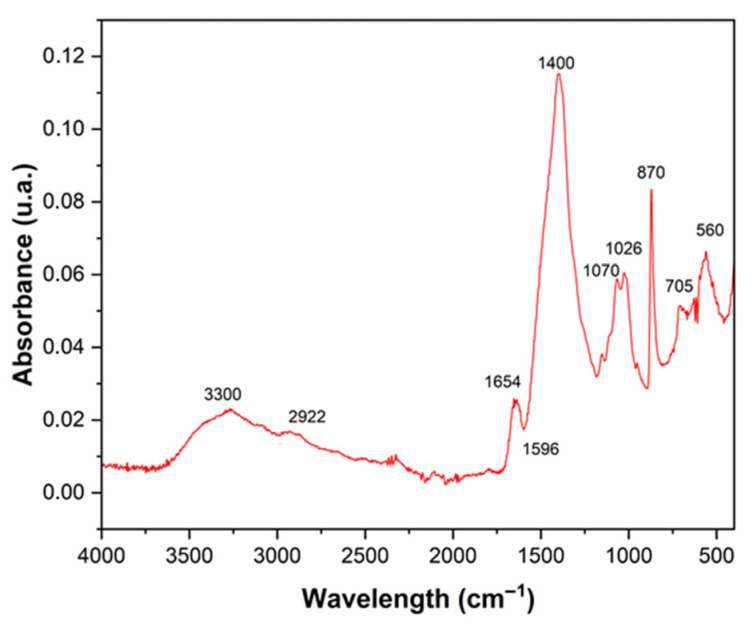
Infrared spectroscopy of the crab residue powder.

Fourier-transform infrared spectroscopy (FT-IR) revealed, in the powder obtained from crab shell, characteristic bands indicative of the presence of functional organic and mineral compounds (Figure 4). The band located at 3300 cm^−1^ is associated with O–H stretching vibrations originating from hydroxyl groups linked to absorbed water [32]. The absorption band at 2922 cm^−1^ is attributed to C–H stretching of aliphatic chains, suggesting the presence of organic compounds such as lipid residues and proteins. In the region of 1654 cm^−1^, a characteristic Amide I band is observed, corresponding to C=O stretching in structural proteins, while the peak at 1596 cm^−1^ is related to Amide II, associated with N–H bending and C–N stretching, both indicating the presence of chitin and protein components in the material matrix [33]. The intense band at 1400 cm^−1^ is attributed to the deformation of methyl (CH_3_) and methylene (CH_2_) groups, typical of the chitin backbone. The bands located at 1070 cm^−1^ and 1028 cm^−1^ are associated with C–O and C–O–C stretching vibrations, characteristic of glycosidic linkages in polysaccharides, corroborating the chitin–carbohydrate composition of the material [34].

Finally, the peaks between 870 and 560 cm^−1^ are associated with deformation vibrations of the carbonate ion (CO_3_^2−^), conforming the presence of calcium carbonate [35], the main mineral component of crab waste, also evidenced in the EDX results.

#### 3.1.3. Correlation Among the Physical Properties of the Powder (Hygroscopicity, True Density and Particle Size)

In Table 1, the correlation coefficients between the physical properties of the crab-residue powder are presented. Particle size showed a moderately strong negative correlation with true density (r = −0.651), while particle size and hygroscopicity exhibited a moderately strong positive correlation (r = 0.610). However, despite these moderately strong correlations, neither result was statistically significant (*p* > 0.05), indicating a lack of evidence for a linear association between those variables. The correlation between hygroscopicity and true density was weak and also non-significant (r = −0.159; *p* > 0.05), indicating absence of correlation between these variables.

These results suggest that, under the conditions of this study, the data do not provide reliable evidence of a linear relationship—underscoring the need for future investigation using a larger dataset. This finding may be explained by the heterogeneity of the powder and granulometric variation, which result in differences in organic–mineral density, irregular particle distribution and may have caused high data dispersion. (This heterogeneity is discussed and illustrated in the SEM analysis.) Moreover, hygroscopicity strongly depends on the surface characteristics of the particles, presence of pores, surface roughness, specific surface area for interaction with water vapor, as well as on structural and morphological factors, which are often more relevant than the bulk (true) density [36].

Moreover, hygroscopicity of the crab powder showed an average value of 1.765 ± 0.3805%, which suggests a low moisture-retention capacity. The experimentally determined mean true density was 2.1147 g·cm^−3^. The mean particle size corresponding to the granulometry used for film fabrication and characterization was 0.12 ± 0.074 mm.

#### 3.1.4. X-Ray Diffraction (XRD)

**Figure 5:**
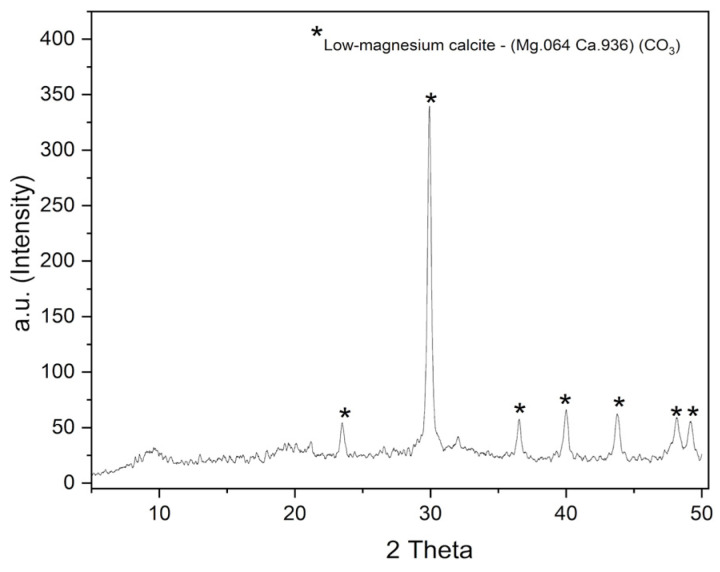
X-ray diffraction pattern of the Uçá crab powder showing characteristic peaks of low-magnesium calcite.

The X-ray diffraction (XRD) analysis indicated that the material exhibits a high-degree crystalline structure, with sharp diffraction peaks characteristic of low-magnesium calcite, according to the standard file of the Joint Committee on Powder Diffraction Files (JCPDS 86-2335) (Figure 5). This result is consistent with the mineralogical analysis by EDX (≈86% Ca and 4.9% Mg), which confirms the incorporation of magnesium into the crystalline lattice, while preserving the low-Mg calcite structure. According to the literature, the incorporation of Mg into calcite modifies its crystal lattice and can influence the hardness and stiffness of the composite [37]. This may affect the mechanical and barrier properties of the films produced with this type of material, since highly crystalline materials tend to be more rigid, harder, and more resistant, making them an ideal choice to be used as structural-reinforcing elements in composites [38]. Therefore, the addition of this powder as a mineral filler in biodegradable films is justified by its crystalline structure and mineral profile, which are compatible with improved mechanical and barrier properties.

#### 3.1.5. Energy-Dispersive X-Ray Spectroscopy (EDX) and Absolute Mineral Content

The analyses revealed that the powder is composed primarily of calcium (Ca), with an absolute content of 22.52 g·kg^−1^, indicating that calcium carbonate (CaCO_3_) is the predominant mineral in the crab residue (Table 2). In addition to calcium, sodium, potassium, magnesium and phosphorus were detected at lower absolute levels (11.11, 6.29, 1.89 and 0.68 g·kg^−1^, respectively), suggesting the presence of phosphates and marine-derived mineral ions retained in the crab’s structural matrix. Other trace elements were also identified, including silicon (Si), strontium (Sr), iron (Fe), copper (Cu), bromine (Br), zinc (Zn) and manganese (Mn). These same elements were also reported by Pessoa et al. [32] in studies of *Ucides cordatus* residues.

The experimentally determined true density of 2.1147 g·cm^−3^ in this work is consistent with the mineral composition detailed from the EDX analysis and the absolute mineral content. It is known that minerals like calcium carbonate (CaCO_3_) have relatively high densities [39]. The organized arrangement of atoms, which leads to a uniform and highly crystalline structure, also raises density values, as shown by the XRD measurements.

#### 3.1.6. Particle Size

**Figure 6:**
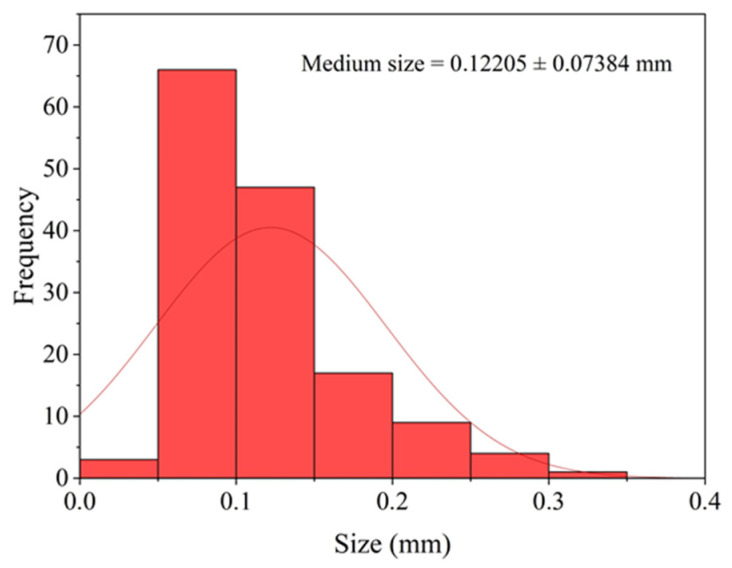
Distribution plot and mean particle size of the powder used in film production.

The mean particle size corresponding to the granulometric distribution used in the film formulation was 0.12 ± 0.074 mm (see Figure 6). The histogram shows that most particles are concentrated within small to intermediate size ranges, with only a few occurrences in the larger size classes.

### 3.2. Film Characterization Results 

#### 3.2.1. Thickness, Solubility and WVP

Thickness is one of the main variables influencing the mechanical and barrier properties of polymeric films [40]. In the present study, the films exhibited thickness values ranging from 0.082 ± 0.0023 to 0.072 ± 0.0024 mm, and the differences between the control treatment (0%) and the films containing 1% and 2% of added powder were statistically significant (Table 3). It was observed that the addition of the powder did not increase the film thickness; instead, a slight reduction was detected. A possible explanation for this behavior is that the incorporation of powder fillers into the films tends to decrease the free volume and increase structural compaction, leading to denser and consequently thinner films [41]. A similar phenomenon was identified by Fu et al. [42], who observed that the inclusion of fillers in the polymer matrix reduced the free volume, resulting in greater compaction and higher structural density of the polymer. The reduction in free space between chains can lead to substantial changes in all film characteristics.

Regarding permeability, the incorporation of microparticles at concentrations of 1% and 2% resulted in a significant reduction in WVP values (Table 3), reaching 0.089 g·mm/m^2^·h·kPa, which corresponds to an approximately 15% decrease compared with the control treatment. This demonstrates that the addition of the powder contributed to the formation of a denser and less permeable matrix. The observed reduction in permeability at these filler concentrations is attributed to the homogeneous dispersion of the powder within the polymer matrix, since rigid fillers that are well distributed help reduce the mobility of adjacent polymer chains, decrease matrix swelling and water uptake, and consequently lower water vapor permeability (WVP) [43]. Furthermore, when particles of high density and rigidity are introduced into the polymer matrix, they act as significant physical barriers to the diffusion of water molecules, resulting in a denser and more compact film, thereby impacting the reduction in water vapor permeability (WVP) [44,45]. This outcome is supported by SEM analysis, which shows the presence of a smooth surface and a compact structure without defects, cracks, or discontinuities for the films containing 1% and 2% powder. A similar effect was reported by Cabrera-Barjas et al. [46] who, upon adding chitin nanofibers as a structural ingredient in pectin films, also reported a significant reduction in WVP values compared with the control.

On the other hand, higher filler concentrations 4% (0.116 g·mm/m^2^·h·kPa) and 5% (0.114 g·mm/m^2^·h·kPa) produced WVP values statistically similar to the control treatment but significantly higher than those obtained with 1%, 2%, and 3% powder. The quadratic regression model (Y = 0.108254 − 0.033360X + 0.015099X^2^; R^2^ = 70.62%) supported this observation, demonstrating a nonlinear relationship between microparticle content and WVP. This means that the amount of filler has two effects on permeability: at first, it makes the barrier better by lowering WVP to a certain degree, but beyond that, more filler starts to weaken the barrier and raise WVP. This behavior is attributed to structural changes in the film, such as an increase in porosity and the formation of preferential pathways for vapor diffusion due to structural flaws, cracks, and discontinuities, which compromise barrier efficiency. These results are supported by SEM analysis, where the micrographs show that the higher powder loadings (4% and 5%) critically and negatively altered the structure, thereby influencing the barrier properties observed in this study. Cabrera-Barjas et al. [13] reported a similar trend, noting that increasing nanochitin loading (10% and 20%) in hydroxypropylmethylcellulose-based films raised WVP values due to pore formation and structural defects caused by excessive filler incorporation into the matrix.

In this study, film solubility increased significantly with the addition of powder, particularly at concentrations of 3%, 4%, and 5% (0.037, 0.037, and 0.038%, respectively) (Table 3). The adjusted linear regression equation, Y = 0.027913 + 0.002736X (R^2^ = 70.40%; *p* < 0.05), indicates that for every 1% increase in powder concentration, solubility increases by approximately 0.0027%. These results demonstrate a load-dependent effect: the higher the filler content, the greater the water absorption and, consequently, the higher the solubility of the film. This increase may be associated with enhanced porosity in films with higher filler content, which likely facilitated moisture uptake by the polymeric matrix. This effect helps maintain a hydrated area while weakening the structure, making it more soluble. This change in structure with the added filler was evidenced by SEM analysis of the films, showing the presence of pores as well as failures, cracks, and discontinuities in the internal structure of the films at the higher percentages (3, 4, and 5%).

Similar results were reported by Mourak et al. [47], in which chitosan films reinforced with different minerals showed increased porosity and water absorption with rising mineral load. Moreover, the results obtained in this study indicate a strong, positive, and significant correlation between solubility and WVP (Table 4). In practical terms, the increase in WVP was directly associated with increased solubility. This suggests that these properties may share structural determinants within the films, such as the presence of porosity, which justifies the observed relationship between these variables.

#### 3.2.2. Color Attributes the Films

**Figure 7:**
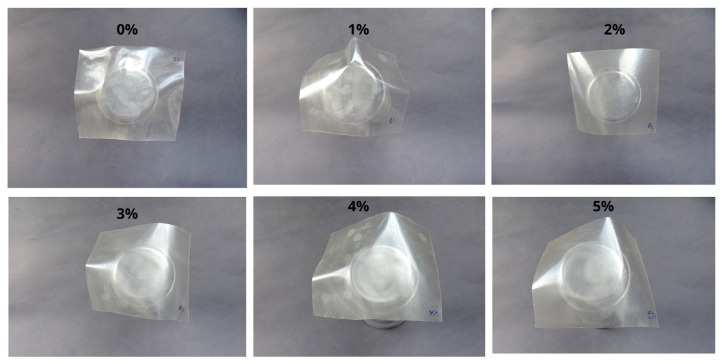
Photographic images of the films containing different powder percentages.

The results showed that the lightness (L*) of the films was significantly affected by the different powder concentrations (Table 5 and Figure 7). This significant difference was observed in the control treatment, which exhibited the highest lightness values (95.540) compared with the treatments containing 4% (94.708) and 5% (94.573) powder. The incorporation of the powder, particularly at higher concentrations, resulted in a significant decrease in lightness, affecting light reflection and making the films less luminous than the control. Regarding the Hue angle (h°), statistically significant differences were mainly observed between the control (89.130°) and the treatments containing 4% and 5% powder (89.367° and 89.403°, respectively). In contrast, intermediate concentrations (2% and 3%) did not differ statistically from the control, indicating that film hue shifts only subtly with varying amounts of added powder, and that such differences become significant only at higher filler levels (4% and 5%).

For chroma (C*), which indicates color vividness or intensity, the 5% treatment (C* = 1.949) stood out as much more saturated compared with the control (C* = 0.727). This variation suggests that higher concentrations of crab powder intensify the coloration of the films, making them more vivid and colorful. This reinforces the idea that saturation increases significantly, but not linearly with rising powder content. It suggests a progressive saturation effect, where higher levels of powder result in more pronounced increases in color intensity.

This behavior may be explained by the direct influence of the powder’s intrinsic color parameters [5], which showed a naturally darker coloration (L = 62.320 ± 1.170), with a yellowish-reddish hue angle (H = 68.415 ± 0.260) and high chroma (C = 13.107 ± 0.423), indicating strong color intensity and saturation (Table 5). Color is a key attribute that can affect consumer perception and acceptance, especially in food and packaging applications, where visual appeal plays a crucial role and can enhance product marketability. Additionally, color parameters can provide insights into chemical interactions and compatibility among film components [48].

The films used for the photographic images were samples measuring 6.5 × 8.0 cm (6.5 cm in width and 8 cm in length).

#### 3.2.3. Mechanical Properties

Table 6 presents the results for tensile strength, ductility, toughness, and elastic modulus of the films produced. The control film, without powder incorporation, exhibited a tensile strength of 7.586 MPa. According to the statistical analysis, no significant differences were observed between the treatments containing 1%, 2%, and 3% powder and the control, indicating that low filler concentrations did not exert a meaningful effect on mechanical resistance. In contrast, the treatments with 4% and 5% powder promoted a significant increase in tensile strength, resulting in increments of 55.34% and 56.78%, respectively, compared with the control. This indicates that the incorporation of the powder provided structural reinforcement to the polymeric matrix, particularly at higher concentrations, making the films more resistant to tensile stress. A similar result was reported by Janik et al. [8], who observed that adding calcium carbonate to a chitosan matrix significantly increased tensile strength, reaching 75 MPa with 5% filler, roughly three times higher than the control. The authors also noted that filler contents between 0.5% and 3% had no significant effect on this property. Another study using carbonated hydroxyapatite in pectin-based films achieved improved tensile strength at higher loadings (5% and 7%), with values of 0.0138 and 0.0153 MPa, respectively [49]. These varied numerical outcomes found in the present study compared to literature are due to the fact that changes in mechanical properties depend not only on the percentage of filler added, but also on the type, shape, and particle size of the filler [8]. Conversely, studies evaluating films composed exclusively of pectin or incorporating less rigid additives have reported tensile strength values lower than those observed in the present study. Alves et al. [22], in developing biodegradable pectin-based films reinforced with microalgae, reported an average tensile strength of approximately 0.0458 MPa. Similarly, Nastasi, Fitzgerald and Kontogiorgos [50] investigated pectin films enriched with fruit extracts rich in polyphenols and reported rupture stress values ranging from 4.99 to 6.91 MPa. These findings demonstrate that, in the absence of mineral reinforcements, the polymeric matrix exhibits limited mechanical strength, a condition that is significantly improved with the incorporation of well-dispersed mineral fillers, as reported in the present study.

The treatments with 4% and 5% powder exhibited, through SEM micrographs, a pronounced microstructural heterogeneity, including particle agglomerations, interconnected pores, cracks, and internal discontinuities. Interestingly, the tensile strength values were higher than those of the control. This result is divergent compared to other works in the literature, such as Tazibt et al. [51], who observed structural defects in composites with increasing hydroxyapatite content that led to a reduction in tensile strength in the presence of such defects. Although microstructural defects are generally associated with decreased mechanical performance in composites, the increase in tensile strength observed for the 4% and 5% powder treatments can be attributed to the mechanical contribution of the rigid mineral particles and their interaction with the polymer matrix. The presence of rigid phases that adhere well to the surrounding matrix restricts the mobility of the polymer chains, resulting in an increased elastic modulus of the composite, which in turn enhances load transfer and raises the capacity to withstand applied stresses prior to fracture. This occurs when the positive effect of particle stiffness and strong particle–matrix interaction outweighs the negative impact of microstructural defects, leading to a trade-off in which stiffness and tensile strength are enhanced, despite the heterogeneity visible in the SEM micrographs [52,53,54]. As evidence of this, Fu et al. [55] state that the mechanical properties of composites composed of particles and polymers are strongly related to particle size, particle–matrix interfacial adhesion, and filler loading. Accordingly, the improvement in tensile strength is related to the intrinsic characteristics of the added material and is associated with good interfacial adhesion between the particles and the polymer matrix, favored by the morphology and texture of the particles, which may have improved this particle–matrix interfacial connection, as discussed in the SEM analysis of the powder.

Ductility showed an inverse behavior compared with tensile strength. The control treatment (12.96%) exhibited the highest elongation before fracture, while increasing powder content led to a significant reduction in deformation capacity, reaching 3.720% and 4.656% for the 4% and 5% treatments, respectively. The linear regression equation (Y = 10.79 − 1.52X; R^2^ = 72.04%) indicates that ductility decreases as powder concentration increases; for each 1% increment in filler, ductility decreases by approximately 1.52%. This demonstrates that powder incorporation restricts the mobility of polymer chains, resulting in films with greater rigidity and lower flexibility, reducing elongation before rupture. This mechanical balance between strength and elongation is characteristic of polymer composites, where increased stiffness and mechanical strength tend to reduce ductility [56]. This result is consistent with the values obtained for the elastic modulus, which increased significantly with higher filler concentrations—4% and 5% (0.160 GPa and 0.147 GPa, respectively). The linear regression model (Y = 0.058214 + 0.018714X; R^2^ = 75.32%) confirms this trend, suggesting that for each 1% increase in powder content, the film’s elastic modulus increases by approximately 0.0187 GPa.

The increase in modulus can be explained by the intrinsic characteristics of the added filler. As evidenced by the EDX and XRD analyses, the powder has a high mineral load, predominantly CaCO_3_. This compound provides rigidity and density to the polymeric matrix, resulting in higher elastic modulus values and increased film stiffness—factors that also influence other mechanical properties such as ductility, toughness, and tensile strength. These findings are supported by Post et al. [57], who observed a significant increase in elastic modulus when adding a predominantly CaCO_3_-based mineral filler to polybutylene succinate, poly(hydroxybutyrate-cohydroxyhexanoate), polybutylene succinate adipate, and polybutylene adipate terephthalate matrices. This reinforcement not only increased stiffness but also affected other properties, including tensile strength, ductility, and toughness behavior similar to that observed in the present study. Similarly, Zaccone et al. [58] reported that the addition of highly crystalline nanochitin also influences the mechanical properties of composites. The authors found that adding 5% nanochitin to a poly(hydroxybutyrate) biopolymer increased tensile strength from 24 MPa to 33 MPa, demonstrating a significant enhancement in mechanical performance, consistent with the reinforcing effects observed in this work.

Toughness is the measure of how much energy a material can absorb before failing, which means that in composite materials, a balance between strength, stiffness, and plastic deformation is essential [59]. As for toughness, the treatments with the powder saw a marked decrease, especially at concentrations ranging from 2% to 5%. The control treatment showed a toughness of 0.104 J, which is statistically similar to the 1% powder treatment at 0.120 J. However, in the treatments with higher percentages of the powder (2 to 5%), the toughness decreased significantly compared to the 0% and 1% treatments. This reduction in toughness at higher powder concentrations can be attributed to the typical effect of rigid mineral fillers such as CaCO_3_, which increase the modulus and stiffness of the material while simultaneously restricting the polymer chain mobility, thereby reducing the film’s energy-absorption capacity [60]. The regression equation Y = 0.116429 − 0.032443X + 0.003929X^2^ (R^2^ = 65.35%) illustrates that with the addition of filler, toughness decreases initially due to the linear rise in powder content, but at greater levels of inclusion, the positive quadratic term suggests a minor mitigation of this reduction. Basically, every 1% more filler takes 0.032443 J off toughness, until the quadratic curve (0.003929X^2^) kicks in and starts to slow that loss or even reverse it a bit at really high concentrations. This behavior correlates with the decrease in ductility that occurs as powder concentration rises. On the other hand, the 1% powder treatment maintained enough flexibility to accommodate deformation and absorb energy before failing, exhibiting a ductility of 8.144%, which was not significantly different from the control’s 12.965%. The energy absorbed until fracture decreased with increasing powder content, inversely to the tensile strength, indicating that the incorporation of mineral particles enhanced the stiffness of the film while reducing its deformation capacity. This behavior is consistent with what has been observed in reinforced polymeric composites, such as chitin films extracted from crab shells and reinforced with coconut shell powder, as reported by Seenuvasan, Malar and Growther [61], in which the addition of reinforcements increased the Young’s modulus while limiting the deformation of the polymeric matrix. This pattern, greater stiffness accompanied by reduced deformability, is typical of reinforced composites and aligns with the mechanical behavior observed in the present study. To sum up, the addition of the powder greatly influenced the mechanical behavior of the films: its considerable rigidity raised both the elastic modulus and tensile strength, but diminished ductility and toughness, particularly at elevated filler levels.

In food packaging, the enhanced rigidity and mechanical strength of the film can be extremely beneficial for preserving the food’s structural integrity during transportation and storage, reducing the chances of physical damage, tears, or punctures [62,63]. Moreover, stiffer and denser films can serve as more efficient barriers to gas diffusion and moisture permeation, which is vital for maintaining the quality, freshness, and safety of food, especially for items that are sensitive to oxygen exposure or moisture loss [62,64].

However, the reduction in ductility that comes with higher filler content could hinder the film’s ability to conform in scenarios where flexibility is essential, like when packaging products with irregular shapes or those that need to fit snugly. When flexibility is reduced, it can lead to cracking, sealing issues, or challenges in the packaging process, which can compromise packaging efficiency and the performance of the final product [65]. It is, therefore, crucial to achieve a balance between rigidity and flexibility in packaging systems that incorporate mineral fillers to ensure not just mechanical protection but also the film’s adaptability to the unique features of the food being packaged.

#### 3.2.4. Scanning Electron Microscopy (SEM)

**Figure 8:**
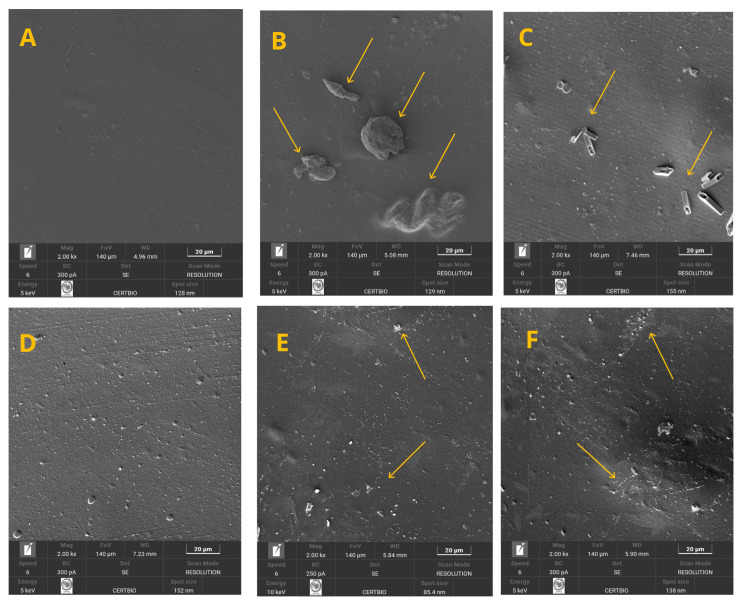
(**A**–**F**) Surface micrographs of the treatments of the 0–5% crab shell powder.

The analyses of the flat surface of the films through scanning electron microscopy (SEM) images are presented in Figure 8A–F. The images show the effect of powder addition at concentrations ranging from 0 to 5%, respectively. By examining Figure 8A–F, it can be observed that increasing the powder concentration in the polymer matrix enhanced the dispersion of the particles. When working with lower proportions, such as 1%, for example, the powder particles appear as agglomerates distributed with larger spacing between them on the surface (Figure 8B). In contrast, the gradual increase in this component promoted greater uniformity in particle distribution (Figure 8C–F).

**Figure 9:**
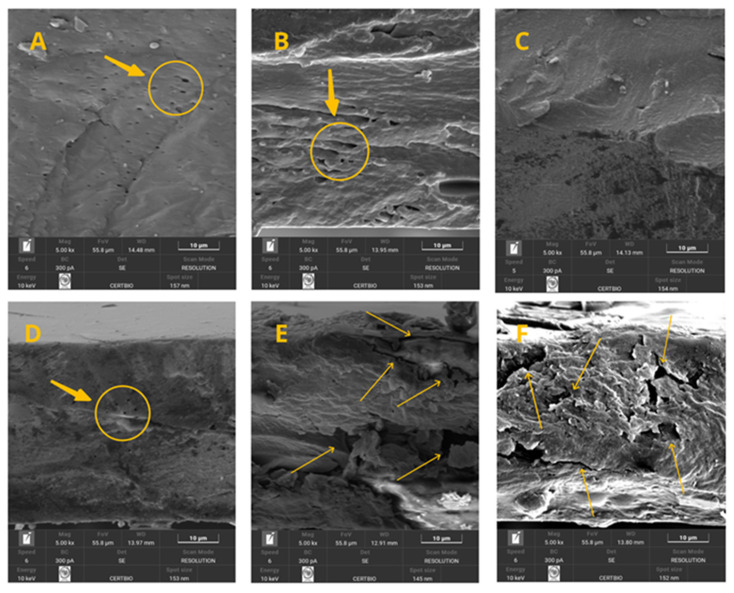
(**A**–**F**) Cross-section micrographs of the treatments of the 0–5% crab shell powder.

Figure 9 presents the cross-sectional images of the film. By analyzing the images (Figure 9A–F), it can be observed that the gradual increase in powder concentration imparted greater stiffness to the polymer matrix and resulted in larger porous spaces. This effect indicates that the film retains flexibility when concentrations of 0, 1, 2, and up to 3% are used (Figure 9A–D). However, at concentrations of 4 and 5% (Figure 9E,F), the material becomes stiffer, which in practice may lead to a more brittle structure with reduced plasticity and elasticity.

These results demonstrate that the amount of filler incorporated into polymer matrices has a significant impact on the morphology and physicomechanical properties of the films [13]. According to Tazibt et al. [51], optimal filler contents can enhance interfacial bonding between the matrix and reinforcing particles. The same authors confirmed this in studies on polymer composites reinforced with hydroxyapatite (HAp) particles, showing that at low filler concentrations, the morphology remained relatively homogeneous and well dispersed, whereas higher concentrations resulted in morphological defects. Likewise, studies on high-density polyethylene (HDPE) composites with hybrid filler systems have shown that composite morphology and mechanical properties can change drastically depending on filler content [66].

## 4. Conclusions

This study demonstrates that the mineral powder obtained from *Ucides cordatus* shells acts as an effective reinforcement for pectin-based biodegradable films. Low filler concentrations (1–3%) were identified as the optimal incorporation range, ensuring the preservation of the matrix’s structural integrity while enhancing both mechanical and barrier properties. In contrast, higher loadings (4–5%) increased film porosity and structural heterogeneity, resulting in embrittlement and reduced overall performance.

The results highlight the relevance of simultaneously considering the nature and the amount of mineral reinforcement when designing polymeric composites. Moreover, the successful use of a low-cost, abundant crustacean by product underscores its potential for valorization in the development of biodegradable materials with tunable properties. Collectively, these findings contribute to advancing sustainable composite design and offer clear guidelines for optimizing formulations of pectin/essential-oil-based polymeric films reinforced with mineral fillers.

## 5. Patents

The patent entitled “Biodegradable film for food preservation based on crab powder and moringa seed oil”, registered under number BR 10 2025 014131 0, is directly related to the present work, differing only in the type of oil employed. Unlike moringa oil, which was used in the patented formulation, this study incorporated pomegranate seed oil (*Punica granatum* L.), as it is far more extensively documented in the scientific literature, particularly regarding its antioxidant and antimicrobial properties, which reinforces its suitability for use in biodegradable systems aimed at food preservation.

## Figures and Tables

**Table 1:** Pearson correlation coefficients of the physical properties of the crab powder.

Variables	Particle Size	True Density	Hygroscopicity
Particle size	1.000	−0.651 ^ns^	0.610 ^ns^
True density	−0.651 ^ns^	1.000	−0.159 ^ns^
Hygroscopicity	0.610 ^ns^	−0.159 ^ns^	1.000

**Table 2:** Qualitative and quantitative elemental composition of the crab residue powder.

Element	Percentage (%)	Absolute Content	Unit
Ca	86.007	22.52	g·kg^−1^
Mg	4.974	1.89	g·kg^−1^
P	3.547	0.68	g·kg^−1^
S	1.877	--	--
Si	1.590	--	--
Sr	1.191	--	--
K	0.623	6.29	g·kg^−1^
Fe	0.136	240.77	mg·kg^−1^
Cu	0.023	18.27	mg·kg^−1^
Br	0.021	--	--
Zn	--	38.72	mg·kg^−1^
Mn	--	5.25	mg·kg^−1^
Na	--	11.11	g·kg^−1^

**Table 3:** Thickness, solubility, and WVP of polymeric films as a function of increasing concentrations of crab residue powder used as a structural filler.

Concentrations (%)	Thickness (mm)	Solubility (%)	WVP (g·mm/m^2^·h·kPa)
0	* 0.082 ± 0.0023 ^a^	* 0.026 ± 0.0056 ^b^	* 0.105 ± 0.0020 ^ab^
1	0.070 ± 0.0062 ^b^	0.029 ± 0.0046 ^ab^	0.089 ± 0.0079 ^c^
2	0.070 ± 0.0017 ^b^	0.034 ± 0.0034 ^ab^	0.089 ± 0.0034 ^c^
3	0.075 ± 0.0033 ^ab^	0.037 ± 0.0030 ^a^	0.094 ± 0.0055 ^bc^
4	0.072 ± 0.0047 ^ab^	0.037 ± 0.0039 ^a^	0.116 ± 0.0077 ^a^
5	0.072 ± 0.0024 ^ab^	0.038 ± 0.0048 ^a^	0.114 ± 0.0031 ^a^
Eq.	-	Y = 0.027913 + 0.002736X	Y = 0.108254 − 0.033360X + 0.015099X^2^
Sign and R^2^	-	(*p* < 0.05; R^2^ = 70.40%)	(*p* < 0.05; R^2^ = 70.62%)
CV (%)	7.32	14.72	6.21

* Values presented as mean ± standard deviation. CV: Coefficient of variation. Eq.: Regression equation. Sign and R^2^: Significance of the beta coefficients and coefficient of determination (R^2^). 0: Film without crab residue powder. 1: Film with 1% powder. 2: Film with 2% powder. 3: Film with 3% powder. 4: Film with 4% powder. 5: Film with 5% powder. Different letters indicate statistically significant differences among treatments according to Tukey’s test (*p* < 0.05).

**Table 4:** Pearson correlation coefficient between WVP and solubility variables.

Variables	r
WVP × Solubility	0.895 **

r: Pearson correlation coefficient. ** Significant at 0.01 (*p* < 0.01). WVP: Water vapor permeability.

**Table 5:** Color parameters L* (lightness), C* (chroma) and H° (hue angle) of the powder and polymeric films as a function of increasing concentrations of crab shell powder used as a structural filler.

Concentrations (%)	L*	H°	C*
Residue powder	* 62.320 ± 1.170	* 68.415 ± 0.260	* 13.107 ± 0.423
0	95.540 ± 0.055 ^a^	89.130 ± 0.094 ^cd^	0.727 ± 0.034 ^d^
1	95.180 ± 0.088 ^ab^	89.424 ± 0.041 ^a^	1.246 ± 0.031 ^c^
2	94.918 ± 0.119 ^bc^	89.256 ± 0.046 ^bc^	1.222 ± 0.070 ^c^
3	94.840 ± 0.102 ^bc^	89.089 ± 0.057 ^d^	0.864 ± 0.038 ^d^
4	94.708 ± 0.286 ^c^	89.367 ± 0.029 ^ab^	1.725 ± 0.099 ^b^
5	94.573 ± 0.185 ^c^	89.403 ± 0.034 ^a^	1.949 ± 0.110 ^a^
CV (%)	0.19	0.07	6.34

* Values are presented as mean ± standard deviation. CV: Coefficient of variation. 0: Film without crab residue powder. 1: Film with 1% powder. 2: Film with 2% powder. 3: Film with 3% powder. 4: Film with 4% powder. 5: Film with 5% powder. L* = Lightness. H° = Hue angle. C* = Chroma. Different letters indicate statistically significant differences between treatments according to Tukey’s test (*p* < 0.05).

**Table 6:** Mechanical properties (tensile strength, ductility, toughness, and elastic modulus) of polymeric films as a function of increasing concentrations of crab shell powder used as a structural filler.

Concentrations (%)	Tensile Strength (Mpa)	Ductility (%)	Toughness (J)	Elastic Modulus (Gpa)
0	* 7.586 ± 0.643 ^b^	* 12.965 ± 3.418 ^a^	* 0.104 ± 0.025 ^a^	* 0.055 ± 0.0030 ^c^
1	11.579 ± 4.225 ^ab^	8.144 ± 3.73 ^b^	0.120 ± 0.011 ^a^	0.095 ± 0.0059 ^b^
2	8.596 ± 1.355 ^ab^	5.382 ± 1.326 ^bcd^	0.048 ± 0.0062 ^b^	0.087 ± 0.0060 ^bc^
3	10.560 ± 3.114 ^ab^	7.158 ± 2.363 ^bc^	0.044 ± 0.012 ^b^	0.085 ± 0.0041 ^bc^
4	11.784 ± 2.537 ^a^	3.720 ± 4.293 ^c^	0.062 ± 0.013 ^b^	0.160 ± 0.025 ^a^
5	11.894 ± 2.937 ^a^	4.656 ± 1.235 ^cd^	0.050 ± 0.017 ^b^	0.147 ± 0.023 ^a^
Eq.	-	Y = 10.79 − 1.52X	Y = 0.116429 − 0.032443X + 0.003929X^2^	Y = 0.058214 + 0.018714X
Sign and R^2^	-	(*p* < 0.05; R^2^ = 72.04%)	(*p* < 0.05; R^2^ = 65.35%)	(*p* < 0.05; R^2^ = 75.32%)
CV (%)	19.99	23.16	24.55	15.95

* Values are presented as mean ± standard deviation. CV: Coefficient of variation. Eq.: Regression equation. Sign and R^2^: Significance of the beta coefficients and coefficient of determination (R^2^). 0: Film without *Ucides cordatus* residue powder. 1: Film with 1% powder. 2: Film with 2% powder. 3: Film with 3% powder. 4: Film with 4% powder. 5: Film with 5% powder. Different letters indicate statistically significant differences among treatments according to Tukey’s test (*p* < 0.05).

## Data Availability

The raw data supporting the conclusions of this article will be made available by the authors on request.
